# Cycloheximide promotes type I collagen maturation mainly via collagen prolyl 4-hydroxylase subunit α2: Cycloheximide promotes type I collagen proline hydroxylation

**DOI:** 10.3724/abbs.2022191

**Published:** 2022-12-20

**Authors:** Run Shi, Miao Xu, Huazhe Ye, Shanshan Gao, Jingfeng Li, Huan Li, Chaoyang Li

**Affiliations:** Affiliated Cancer Hospital & Institute of Guangzhou Medical University State Key Laboratory of Respiratory Disease Guangzhou 510095 China

**Keywords:** cycloheximide, type I collagen, collagen prolyl 4-hydroxylases, proline hydroxylation

## Abstract

Aberrant deposition of collagen is associated with cancer development and tissue fibrosis. Proline hydroxylation, catalyzed by collagen prolyl 4-hydroxylases (C-P4Hs), is necessary for collagen maturation and secretion. Here, we try to evaluate the mechanism of the regulation of CHX on collagen maturation. Using pepsin digestion, liquid chromatograph mass spectrometry and gene knockout, we find that treatment of mouse embryonic fibroblasts with cycloheximide (CHX) increases type I collagen proline hydroxylation partially via P4HA1 and mainly via P4HA2. Western blot analysis results show that CHX treatment reduces type I collagen but does not obviously impact the level of P4HA1/2 protein in the endoplasmic reticulum, which enhances the molar ratio of P4HA1/2 to type I collagen, and coimmunoprecipitation results confirm that more P4HA1/2 can bind to each type I collagen. Since C-P4Hs possess the capability to hydroxylate proline independent of ascorbate for a few cycles, this enhanced binding between P4HA1/2 and type I collagen can partially explain how CHX stimulates type I collagen maturation.

## Introduction

The collagen superfamily comprises 28 members that can be grouped into fibrillar and nonfibrillar collagens [1]. Fibrillar collagens not only provide three-dimensional frameworks for tissues and organs but also make up the main stroma of the tumor microenvironment and play a critical role in cancer progression [ 2– 4] .

As the main fibrillar collagen, type I preprocollagen is translated on ribosomes along the endoplasmic reticulum (ER). Upon entering the ER, the amino-terminal signal is cleaved to form type I procollagen, whose proline and lysine may be hydroxylated by prolyl- and lysyl-hydroxylases.

Proline hydroxylation is an important rate-limiting step in collagen synthesis that is catalyzed by collagen prolyl 4-hydroxylase (C-P4H) [5]. C-P4H is a member of the Fe ^2+^ and α-ketoglutarate-dependent dioxygenase family and exists as α2β2 tetramer; the α-subunit is the catalytic subunit, and the β-subunit is the protein disulfide isomerase. Three isoforms of the α-subunit (P4HA1, P4HA2, and P4HA3) have been identified in humans and mice [5]. Molecular oxygen (O _2_), α-ketoglutarate, and Fe ^2+^ are required for C-P4H activity [5]. Ascorbate is a cofactor of C-P4H and is thought to act as an electron donor that maintains the iron in the Fe ^2+^ state within the C-P4H molecule, thus enabling the enzyme to repeatedly hydroxylate proline residues [6]. A deficiency of ascorbate leads to scurvy, a disease caused by collagen instability [7]. However, C-P4H could catalyze proline hydroxylation at a maximal rate for at least a few cycles in the complete absence of ascorbate [8]. In addition, proline hydroxylation in collagen can also occur under only lactate, peroxidase enzymes, and superoxide treatment [ 9– 11] . The above studies suggest that C-P4Hs could regulate proline hydroxylation in collagen through different mechanisms.

Cycloheximide (CHX), a natural product from the bacterium *Streptomyces griseus*, is a well‐known inhibitor of protein biosynthesis in eukaryotic cells [12]. CHX exerts its effect by binding to the E-site of the 60S ribosomal unit, thus interfering with the function of deacetylated tRNA [ 13– 15] . CHX also stimulates glycogenolysis, gluconeogenesis, and ureogenesis [16]. Furthermore, an earlier study showed that CHX could promote collagen proline hydroxylation [9], but the mechanism was not studied yet.

In this study, we found that CHX reduces type I collagen in the ER of mouse embryonic fibroblasts (MEFs) but does not obviously impact the level of P4HA1/2 protein. The binding of P4HA1/2 to each type I collagen was significantly increased, hence contributing to increased proline hydroxylation in collagen, and P4HA2 has a greater impact on type I collagen maturation than P4HA1.

## Materials and Methods

### Antibodies and reagents

Cycloheximide (CHX) (01810), ascorbate (A7506), actinomycin D (Act D) (50-76-0), pepsin (P7000), and ethyl-3,4-dihydroxybenzoate (EDHB) (E24859) were purchased from Sigma-Aldrich (Darmstadt, Germany). Goat anti-human type I collagen (1310-01) antibody, which was shown to react with mouse type I collagen, was purchased from Southern Biotech (Birmingham, USA). GM130 rabbit polyclonal antibody (A5344), P4HA1 rabbit polyclonal antibody (A3999), HRP-conjugated goat anti-mouse IgG (H+L) (AS003), HRP-conjugated goat anti-rabbit IgG (H+L) (AS014), and HRP-conjugated rabbit anti-goat IgG (H+L) (AS029) were purchased from ABclonal (Boston, USA). P4HA2 rabbit polyclonal antibody (CSB-PA017340ESR1HU) was purchased from Cusabio (Wuhan, China). All antibodies were used according to the instructions of the manufacturer. Proteinase inhibitor cocktail (11697498001) and 4′,6-diamidino-2-phenylindole (DAPI) (10236276001) were purchased from Roche Diagnostics (Mannheim, Germany). Alexa Fluor 488-conjugated donkey anti-goat IgG (ANT082) was purchased from AntGene Biotech (Wuhan, China). Alexa Fluor 555-conjugated donkey anti-rabbit IgG (A21206) was purchased from Invitrogen (Carlsbad, USA). Protein G Agarose Fast Flow beads (16-266) were purchased from Merck Millipore (Darmstadt, Germany). Anti-fade mounting medium (P0126) was purchased from Beyotime Biotechnology (Shanghai, China).

### Collagen production

MEFs (SCRC-1040; ATCC Manassas, USA) were maintained in Dulbecco’s modified Eagle’s medium (DMEM, high glucose; Gibco, Amarillo, USA) supplemented with heat inactivated 10% fetal bovine serum (FBS; HyClone, Logan, USA) and 1% antibiotic penicillin-streptomycin solution (PS; Biological Industries, Kibbutz Beit-Haemek, Israel). *P4ha1* null MEFs, *P4ha2* null MEFs, and *P4ha1*/ *2* double null MEFs were established as reported previously [ 11, 17] and maintained as above. All cells were cultured in an incubator at 37°C with 5% CO _2_ and 95% humidity.

To evaluate the effect of CHX on collagen production, MEFs, *P4ha1* null MEFs, *P4ha2* null MEFs, or *P4ha1*/ *2* double null MEFs were seeded in 6-well plates and cultured for 24 h in DMEM containing 10% FBS and 1% PS until 85% confluence. Cells were then maintained in DMEM containing 1% FBS and 1% PS overnight and stimulated in serum-free DMEM containing different concentrations of CHX or 50 μM ascorbate for 6 h or as indicated in the figure. At the end of treatment, conditioned medium was collected and spun at 1000 *g* for 30 min. Collected media containing type I collagen were precipitated with 176 mg/mL ammonium sulfate for 24 h at 4°C. After precipitation, the samples were centrifuged at 10,000 *g* for 1 h, and the pellet was solubilized in a solution containing 0.4 M NaCl, 0.1 M Tris, pH 7.4.

To determine whether the secreted type I collagen was crosslinked in a triple helix structure and resistant to pepsin digestion, collected media containing type I collagen were precipitated with 176 mg/mL ammonium sulfate as above. The pellets from the precipitated media were resuspended in 10 μM acetic acid and digested with 1 mg/mL pepsin at 4°C overnight.

For western blot analysis of type I collagen in cell lysate, the cell lysate was produced according to Shi *et al*. [17]. Briefly, cell lysates were prepared in lysis buffer containing 20 mM Tris, pH 7.5, 150 mM NaCl, 1 mM EDTA, 1 mM EGTA, 1% Triton X-100, 2.5 mM sodium pyrophosphate, 1 mM β-glycerol phosphate, 1 mM Na _3_VO _4_, 1 mM phenylmethylsulfonyl fluoride (PMSF) and protease inhibitor cocktail and then sonicated at 1 Joule for six seconds on ice. The cell debris was removed by centrifugation, and the protein concentration was quantified using a Bio-Rad Protein Assay Kit II (Bio-Rad, Hercules, USA). The protein concentration of the media was determined according to the protein concentration of the cell lysate.

### Western blot analysis

To determine the level of type I collagen secreted from control and CHX-treated MEFs, western blot analysis was performed on the cell lysate and conditioned media according to Shi *et al*. [17]. The samples were mixed with 4×SDS reducing sample buffer containing 40% glycerol, 250 mM Tris-HCl, pH 6.8, 8% sodium dodecyl sulfate, 0.04% bromophenol blue, and 20% 2-mercaptoethanol. The samples were then heated at 100°C for 10 min and subjected to 7.5% or 10% SDS-PAGE. After electrophoresis, proteins were transferred to a nitrocellulose membrane (HATF00010; Merck Millipore, Billerica, USA), which was blocked with 5% BSA in Tris-buffered saline (TBS) with 0.1% Tween-20 (TBST) at room temperature (RT) for 3 h and then incubated with anti-type I collagen antibody (0.4 μg/mL), anti-P4HA1 (1 μg/mL), anti-P4HA2 (1 μg/mL), or anti-actin antibody (0.2 μg/mL) overnight at 4°C. Bound primary antibodies were detected with HRP-conjugated rabbit anti-goat, goat anti-rabbit or anti-mouse IgG secondary antibodies (0.12 μg/mL). The amount of type I collagen was quantified by densitometry using ImageJ software (NIH) and was normalized to actin loading controls.

### Quantitative real-time PCR (RT-qPCR)

To evaluate the effect of CHX on *Col1α1*, *Col1α2*, *P4ha1* and *P4ha2* mRNA expression, MEFs were grown in DMEM containing 10% FBS and 1% PS in 6-well plates until they reached 85% confluence. The cells were maintained in DMEM containing 1% FBS and 1% PS overnight and treated for 0, 1, 3, and 6 h in serum-free DMEM containing 5 μg/mL CHX. Total RNA was isolated using the RNAprep FastPure Tissue&Cell Kit (Tsingke Biological Technology, Beijing, China). RNA (1 μg) was reverse transcribed using a PrimeScriptTM RT reagent kit (Vazyme, Nanjing, China). RT-qPCR was carried out using SYBR Green Supermix (Bio-Rad) on a Bio-Rad Connect TM real-time PCR system (CFX Connect TM Optics Module; Bio-Rad).

Each 10 μL reaction mixture contained cDNA templates, primer pairs, and SYBR Green Supermix. Amplification occurred after initial denaturation at 95°C for 3 min, followed by 40 cycles at 95°C for 15 s, 53°C for 15 s, and 72°C for 20 s. *Actin* was used as the reference gene. Gene-specific primers were designed according to Shi *et al*. [17] and listed in Table 1.

**Table:** **Table 1** Sequence of primers used in this study

Gene	Primer sequence
*P4ha1*	Forward: 5′-CAAGCAGGAGGACGAGTGG-3′
Reverse: 5′-TGGGTTTGAAATGGTGGC-3′
*P4ha2*	Forward: 5′- GTGTGGACGACTGCTTTGG-3′
Reverse: 5′-TTGACAGTGATTTCCCTCTTTCT-3′
*Col1α1*	Forward: 5′- GAGCGGAGAGTACTGGATCG-3′
Reverse: 5′- TACTCGAACGGGAATCCATC-3′
*Col1α2*	Forward: 5′-GCGGTGAAGAAGGAAAGAGA-3′
Reverse: 5′- CCAGGAGACCCAGGAAGAC-3′
*β-Actin*	Forward: 5′-CGTGCGTGACATCAAAGAGAAGC-3′
Reverse: 5′-TGGATGCCACAGGATTCCATACC-3′

### Inhibitor treatment

To determine whether CHX increases mature type I collagen secretion by regulating its transcription, MEFs were treated with Act D, a DNA transcription inhibitor. MEFs were grown in DMEM containing 10% FBS and 1% PS in 6-well plates until 85% confluence. The cells were maintained in DMEM containing 1% FBS and 1% PS overnight and treated for 6 h in serum-free DMEM containing 5 μg/mL CHX alone or 5 μg/mL CHX plus 2 μg/mL Act D. After treatment, the cell lysate and media were collected and assayed as above.

To determine whether CHX increases type I collagen maturation by regulating the activity of C-P4H, MEFs were treated with EDHB, an inhibitor of C-P4H. MEFs were grown in DMEM containing 10% FBS and 1% PS in 6-well plates until 85% confluence. The cells were maintained in DMEM containing 1% FBS and 1% PS overnight and treated for 6 h in serum-free DMEM containing 5 μg/mL CHX alone or 5 μg/mL CHX plus different concentrations of EDHB as indicated in the figure or supplemental figure, respectively. After treatment, cell lysates and media were collected and assayed as above.

### Immunofluorescence staining

To show that CHX stimulates type I collagen secretion via the Golgi apparatus, MEFs were seeded on poly-D-lysine-coated glass bottom Petri dishes (801002; NEST, Wuxi, China) and cultured for 24 h in DMEM containing 10% FBS and 1% PS until 85% confluence. Cells were then cultured in DMEM containing 1% FBS and 1% PS overnight and stimulated for 6 h in DMEM containing 1% FBS and 1% PS with or without 5 μg/mL CHX. After treatment, indirect immunofluorescence staining was performed to detect the localization of type I collagen according to Shi *et al*. [17]. Briefly, the cells were rinsed twice with PBS and fixed with 4% paraformaldehyde in PBS for 10 min at room temperature (RT). The fixed cells were then permeabilized with 0.1% Triton X-100 in PBST (PBS with 0.1% Tween-20) for 10 min. Permeabilized cells were blocked with 5% BSA in PBST for 2 h at RT. To detect the colocalization of type I collagen and the Golgi apparatus, cells were incubated with goat anti-type I collagen or rabbit anti-GM130 (Golgi apparatus maker) for 2 h at RT. After incubation, the cells were rinsed six times with PBST and then incubated with Alexa Fluor 555-conjugated donkey anti-rabbit or Alexa Fluor 488-conjugated donkey anti-goat IgG for 1 h at RT. After incubation, the nuclei were counterstained with DAPI (500 ng/mL) for 5 min and then rinsed six times with PBST. Finally, the treated cells were covered with antifade mounting medium and observed on an LSM880 confocal laser scanning microscope (Zeiss, Oberkochen, Germany).

### Coimmunoprecipitation (Co-IP)

To detect the interaction between P4HA1/2 and type I collagen in MEFs with or without CHX. MEFs were grown in 10% FBS and 1% PS DMEM until they reached 85% confluence. The cells were then maintained in DMEM containing 1% FBS and 1% PS overnight and treated for 6 h in serum-free DMEM with or without 5 μg/mL CHX. Co-IP was performed according to Shi *et al*. [17]. Briefly, Co-IP was performed using 2 μg of anti-Type I collagen antibody and its corresponding isotype control antibody. Then, 1 mL of cell lysate was incubated with anti-Type I collagen antibody and 40 μL of 50% slurry of protein G Sepharose beads at 4°C overnight. After six times wash with cell lysis buffer, the beads were boiled in 40 μL 2× SDS reducing sample buffer. Finally, 20 μL cell lysates subject to 10% SDS-PAGE.

### Liquid chromatograph mass spectrometer (LC-MS)

To determine whether CHX treatment enhances proline hydroxylation on type I collagen, hydroxyproline on type I collagen was detected via LC-MS according to Shi *et al*. [17]. MEFs cultured in 10 petri dishes (150 mm diameter) were treated with or without CHX. Conditioned media were concentrated using Amicon Ultra15, 30K (UFC 903001; Millipore). The concentrated media were mixed with 4× SDS reducing sample buffer and separated by 7.5% SDS-PAGE. The gel was stained with Coomassie brilliant blue, and slices containing col1α1 and col1α2 were cut out for LC-MS. The peptide solutions were digested with trypsin and analyzed by LC-MS/MS on an Orbitrap Elite mass spectrometer (Thermo Fisher Scientific). Identification of peptides containing proline or hydroxyproline residues was performed by searching the acquired MS/MS spectra against UniProt-Mus (musculus.fasta) using Proteome Discoverer 1.4 software (Thermo Fisher Scientific). The quantification of hydroxyproline in type I collagen was performed using the ratio of the number of hydroxyproline to the number of total peptides.

### Statistical analysis

All experiments were repeated at least three times. Data are expressed as the mean±SEM. A two-tailed *t* test was performed to compare the measurements between the two treatment groups. *P*<0.05 was considered statistically significant.

## Results

### CHX induces type I collagen maturation in MEFs

**Figure 1:**
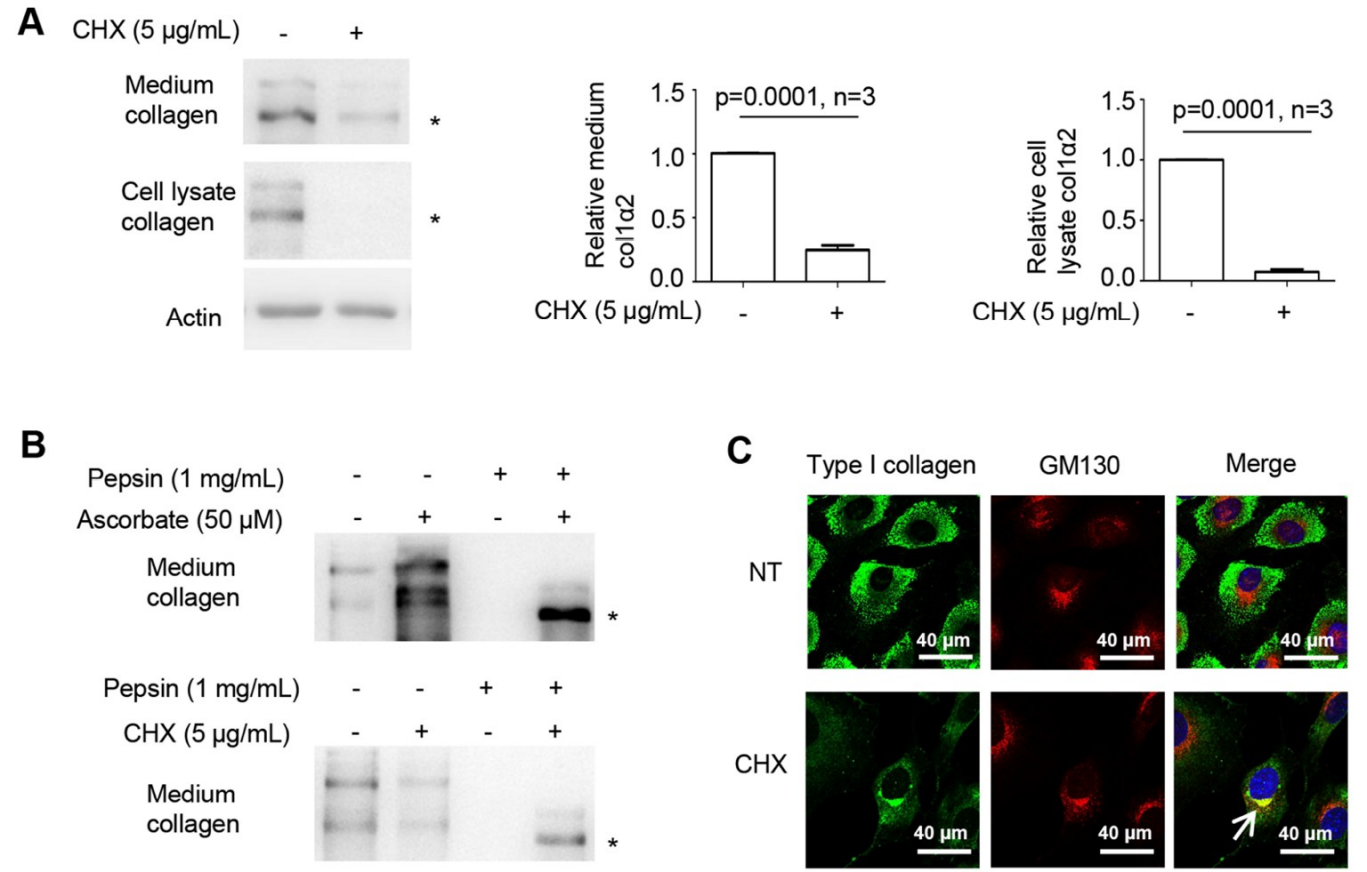
Figure 1 CHX could induce type I collagen maturation (A) Western blot analysis using type I collagen-specific antibody showed that less type I collagen was secreted into the culture medium 6 h after CHX addition. Accordingly, less type I collagen was detected in the cell lysates. (B) Treatment with CHX for 6 h induced pepsin-resistant type I collagen in the culture medium, and ascorbate treatment was used as a positive control. (C) CHX induced type I collagen (green) to enter the Golgi apparatus (red stained by GM130). NT: no CHX treatment. The white arrow points to the colocalization of type I collagen and the Golgi apparatus. col1α2: type I collagen α2; * indicates the position of col1α2 or pepsin-resistant col1α2. Actin was used as the loading control, and the medium and cell lysate levels of col1α2 was quantified by ImageJ software. Statistical analysis was performed from three or four independent experiments as indicated. P values are indicated in each panel if available.

As an inhibitor of protein synthesis, CHX has been shown to induce type I collagen maturation [9]. To investigate the potential mechanism for this phenomenon, we incubated MEFs with or without CHX and detected type I collagen levels in the cell lysate and in the culture medium. The results showed that a residual amount of type I collagen could be detected in the culture medium, while almost no type I collagen could be detected in the cell lysate after CHX treatment for 6 h ( Figure 1A). Type I collagen is composed of two col1α1 and one col1α2. col1α2 but not col1α1, was confirmed to be well detected by the antibody used in a previous study [17]. We thus quantified col1α2 to represent the level of type I collagen. Mature collagen contains a triple helical structure, which is resistant to pepsin digestion. To assess whether CHX-induced type I collagen in the culture medium is mature, type I collagen was treated with pepsin [ 11, 17] . The western blot analysis results showed that the type I collagen in the culture medium was resistant to pepsin digestion, suggesting that CHX indeed stimulated collagen maturation ( Figure 1B, bottom panel). Collagen is secreted into the culture medium through the Golgi apparatus. If CHX induces type I collagen maturation, type I collagen should be detected entering the Golgi apparatus. IFA showed that type I collagen could be detected in the Golgi apparatus after CHX treatment ( Figure 1C). Hence, CHX can stimulate some type I collagen maturation, a result consistent with a previous study [8].

### CHX-induced type I collagen maturation is independent of type I collagen transcription

**Figure 2:**
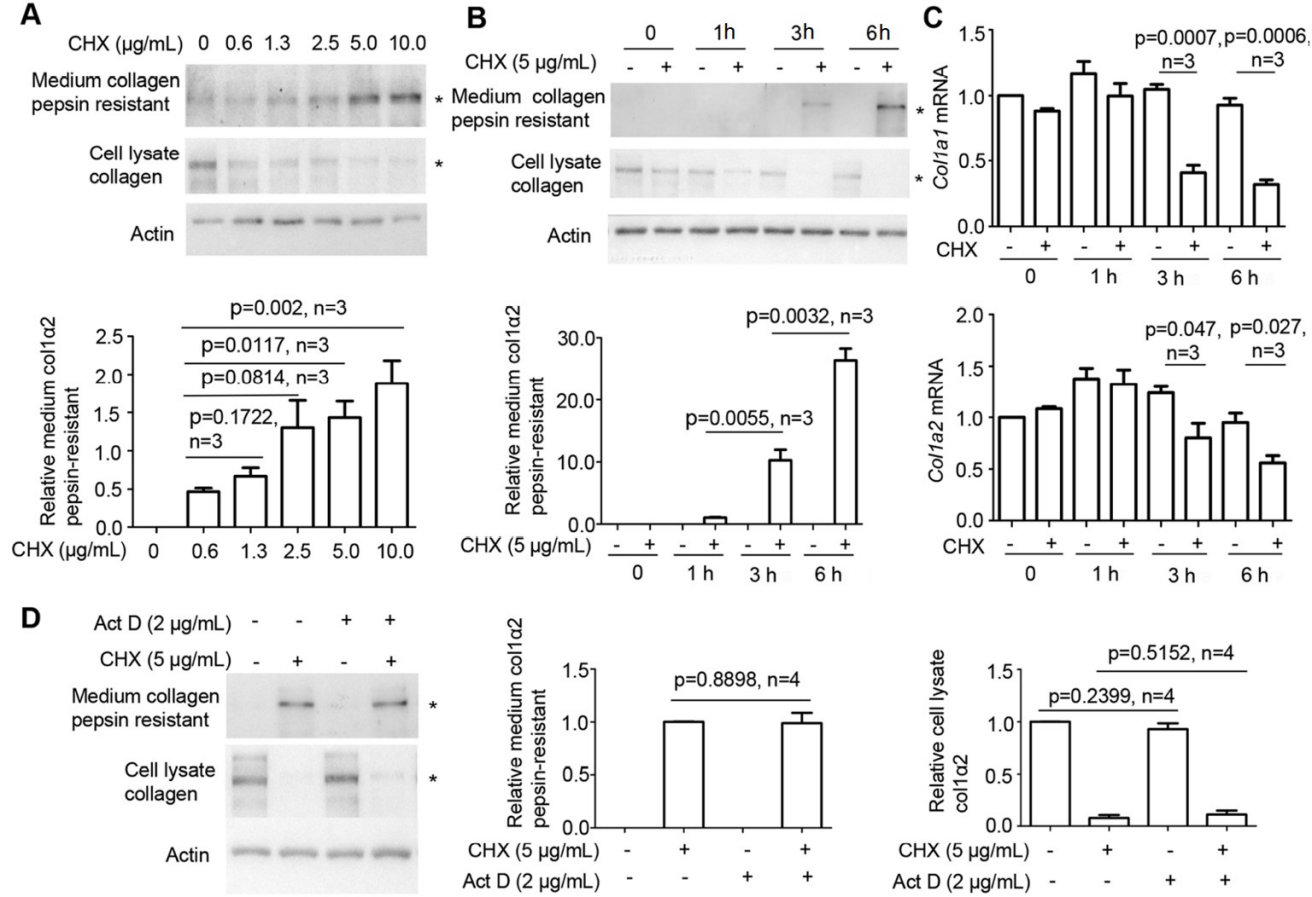
Figure 2 CHX-induced type I collagen maturation is independent of type I collagen transcription (A) Treatment with 5 μg/mL CHX obviously induced detectable pepsin-resistant type I collagen in the culture medium. (B) Pepsin-resistant type I collagen could be detected in the culture medium 3 h after the addition of CHX. (C) CHX stimulation significantly reduced Col1α1 or Col1α2 mRNA levels at 3 and 6 h after treatment as determined by RT-qPCR. (D) Western blot analysis using type I collagen-specific antibody showed that blocking mRNA transcription with Act D did not significantly reduce pepsin-resistant type I collagen in culture medium induced by CHX. col1α2: type I collagen α2; * indicates the position of col1α2 or pepsin-resistant col1α2. Actin was used as the loading control, and medium and cell lysate levels of col1α2 were quantified by ImageJ software. Statistical analysis was performed from three or four independent experiments as indicated. P values are indicated in each panel if available.

We then assessed the minimum concentration of CHX required to induce pepsin-resistant type I collagen in the culture medium. When MEFs were treated with CHX at a concentration of 5 μg/mL for 6 h, obviously more pepsin-resistant type I collagen could be detected in the culture medium ( Figure 2A, upper panel), while type I collagen in the cell lysate was found to be decreased ( Figure 2A, middle panel). We further determined how long the treatment was required for pepsin-resistant type I collagen to be detected in culture medium post CHX treatment. Western blot analysis results showed that more pepsin-resistant type I collagen was detected in the culture medium 3 and 6 h post treatment ( Figure 2B, upper panel), while residual cell lysate type I collagen could be detected at the same timepoints ( Figure 2B, middle panel). Since CHX stimulates type I collagen maturation, we then investigated the effect of CHX on type I collagen transcription. RT-qPCR was performed to quantify the type I collagen mRNA levels from MEFs incubated with or without CHX. The results showed that *Col1α1* and *Col1α2* mRNA levels were decreased after CHX treatment for over 3 h ( Figure 2C). In addition, the addition of Act D to the culture with CHX did not reduce the level of type I collagen in cell lysate or the level of pepsin-resistant type I collagen in the culture medium ( Figure 2D), suggesting that CHX-induced type I collagen maturation is independent of type I collagen transcription.

### CHX induces type I collagen maturation mainly via P4HA2

**Figure 3:**
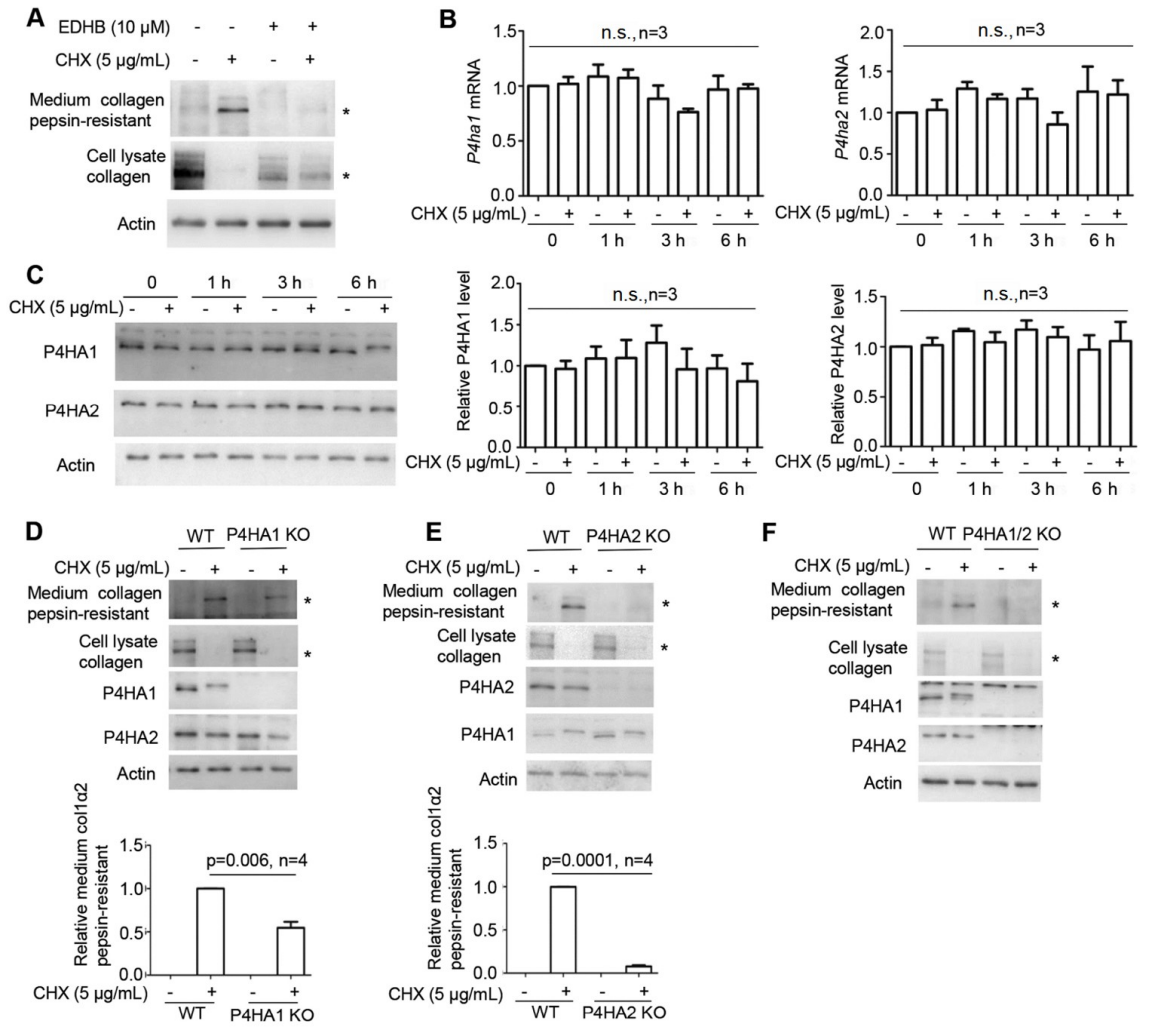
Figure 3 CHX induces type I collagen maturation mainly via P4HA2 (A) Western blot analysis using type I collagen-specific antibody showed that inhibiting C-P4H with EDHB significantly reduced pepsin-resistant type I collagen in culture medium induced by CHX. (B) The addition of CHX did not result in significant differences in P4ha1 or P4ha2 mRNA levels within 6 h as determined by RT-qPCR. (C) Western blot analysis using P4HA1- or P4HA2-specific antibodies showed that CHX did not enhance the levels of these two enzymes. P4HA1/actin or P4HA2/actin was quantified by ImageJ software by arbitrarily defining the signal at 0 h after the addition of CHX as 1. (D–F) Silencing of P4ha1 (D) or P4ha2 (E) decreased the amount of pepsin-resistant type I collagen in the culture medium. Silencing both P4ha1 and P4ha2 (F) completely blocked pepsin-resistant type I collagen in the culture medium. col1α2: type I collagen α2; n.s.: no significance; * indicates the position of col1α2 or pepsin-resistant col1α2. Actin was used as the loading control, and pepsin-resistant col1α2 in the culture medium was quantified by ImageJ software. Statistical analysis was performed from three or four independent experiments as indicated. P values are indicated in each panel if available.

Since CHX stimulates mature type I collagen secretion, we then determined whether CHX-induced type I collagen maturation depends upon C-P4H enzymatic activity. MEFs were treated with EDHB in the presence or absence of CHX. Western blot analysis results showed that CHX-induced mature type I collagen secretion was blocked by EDHB in a dose-dependent manner ( Figure 3A and Supplementary Figure S1), supporting that CHX-induced type I collagen maturation is dependent on C-P4H catalytic activity. Both P4HA1 and P4HA2 are expressed in MEFs [17], and we then investigated whether CHX treatment increases the expression of P4HA1 and P4HA2. Incubation of MEFs with CHX enhanced neither the mRNA levels of *P4ha1* and *P4ha2* ( Figure 3B) nor the protein levels of P4HA1 and P4HA2 ( Figure 3C). Hence, CHX-induced type I collagen maturation is not resulted from increased levels of P4HA1 or P4HA2. To differentiate the roles of these two enzymes in CHX-stimulated type I collagen maturation and secretion, *P4ha1* and/or *P4ha2* were knocked out in MEFs with CRISPR/Cas9 as reported [ 11, 17] . Unexpectedly, silencing of *P4ha2* decreased approximately 95% of pepsin-resistant type I collagen in the culture medium, while silencing of *P4ha1* reduced approximately 40% of pepsin-resistant type I collagen in the culture medium ( Figure 3D,E). When both *P4ha1* and *P4ha2* were silenced, no pepsin-resistant type I collagen was detected in the culture medium upon CHX treatment ( Figure 3F). These results suggested that P4HA2 is the major enzyme involved in CHX-induced type I collagen maturation.

### CHX differs from ascorbate in inducing type I collagen maturation

**Figure 4:**
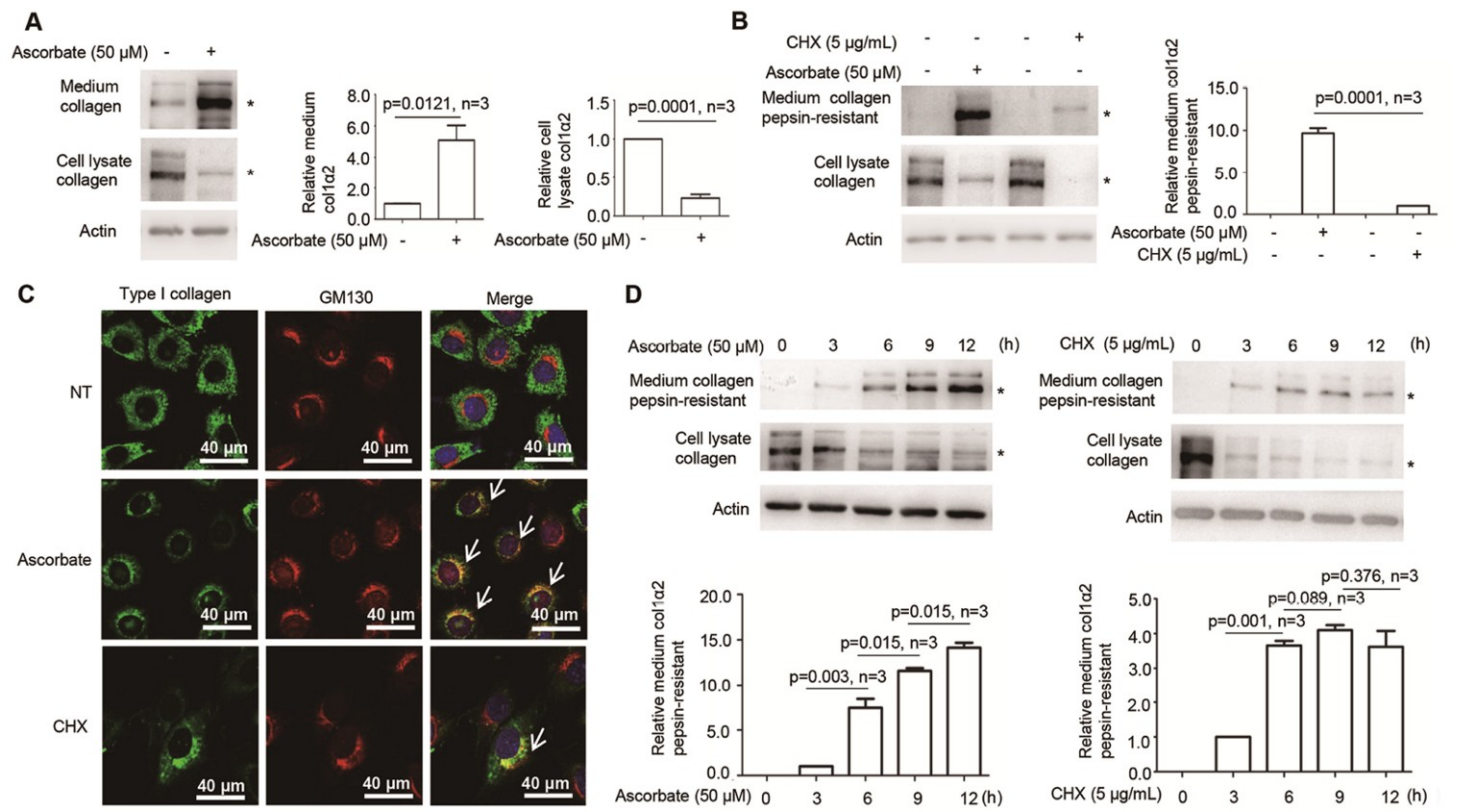
Figure 4 CHX differs from ascorbate in inducing type I collagen maturation (A) Western blot analysis using type I collagen-specific antibody showed that ascorbate increased type I collagen secretion. More type I collagen was secreted into the culture medium 6 h post ascorbate addition. Accordingly, less type I collagen was detected in the cell lysates. (B) Western blot analysis using type I collagen-specific antibody showed that CHX-induced type I collagen was significantly less than that induced by ascorbate. (C) Ascorbate treatment induced more type I collagen (green) to enter the Golgi apparatus (red stained by GM130). Arrows point to the colocalization of type I collagen and the Golgi apparatus. (D) Western blot analysis using type I collagen-specific antibody showed that pepsin-resistant type I collagen levels in the culture medium were saturated at 6 h and remained relatively stable until 12 h post CHX treatment. col1α2: type I collagen α2; * indicates the position of col1α2 or pepsin-resistant col1α2. Actin was used as the loading control, and col1α2 levels in the culture medium and cell lysate were quantified by ImageJ software. Statistical analysis was performed from three independent experiments as indicated. P values are indicated in each panel if available.

Since CHX-induced type I collagen maturation mainly depends upon P4HA2, which differs from that induced by ascorbate [17], we then investigated whether other aspects are different between CHX and ascorbate treatment. After treatment with ascorbate, obviously more type I collagen could be detected in the culture medium ( Figure 4A). In contrast, after treatment with CHX, much less type I collagen could be detected in the culture medium ( Figure 1A). In fact, ascorbate induced 10 times more pepsin-resistant type I collagen in the culture medium than CHX ( Figure 4B). To explore this further, we assessed the amount of type I collagen entering the Golgi apparatus after CHX or ascorbate treatment. A lower colocalization between type I collagen and the Golgi apparatus was observed in CHX-treated MEFs ( Figure 4C).

In addition, the pepsin-resistant type I collagen level in the culture medium was saturated at 6 h and remained relatively stable until 12 h post CHX treatment, whereas ascorbate consistently increased mature type I collagen synthesis from 6 h to 12 h ( Figure 4D). In addition, *Col1α1* and *Col1α2* mRNA levels were significantly decreased in MEFs treated with CHX for 3 or 6 h ( Figure 2C), which was different from those treated with ascorbate [17].

### CHX increases type I collagen prolyl hydroxylation by enhancing the binding between P4HA1/2 and type I collagen

**Figure 5:**
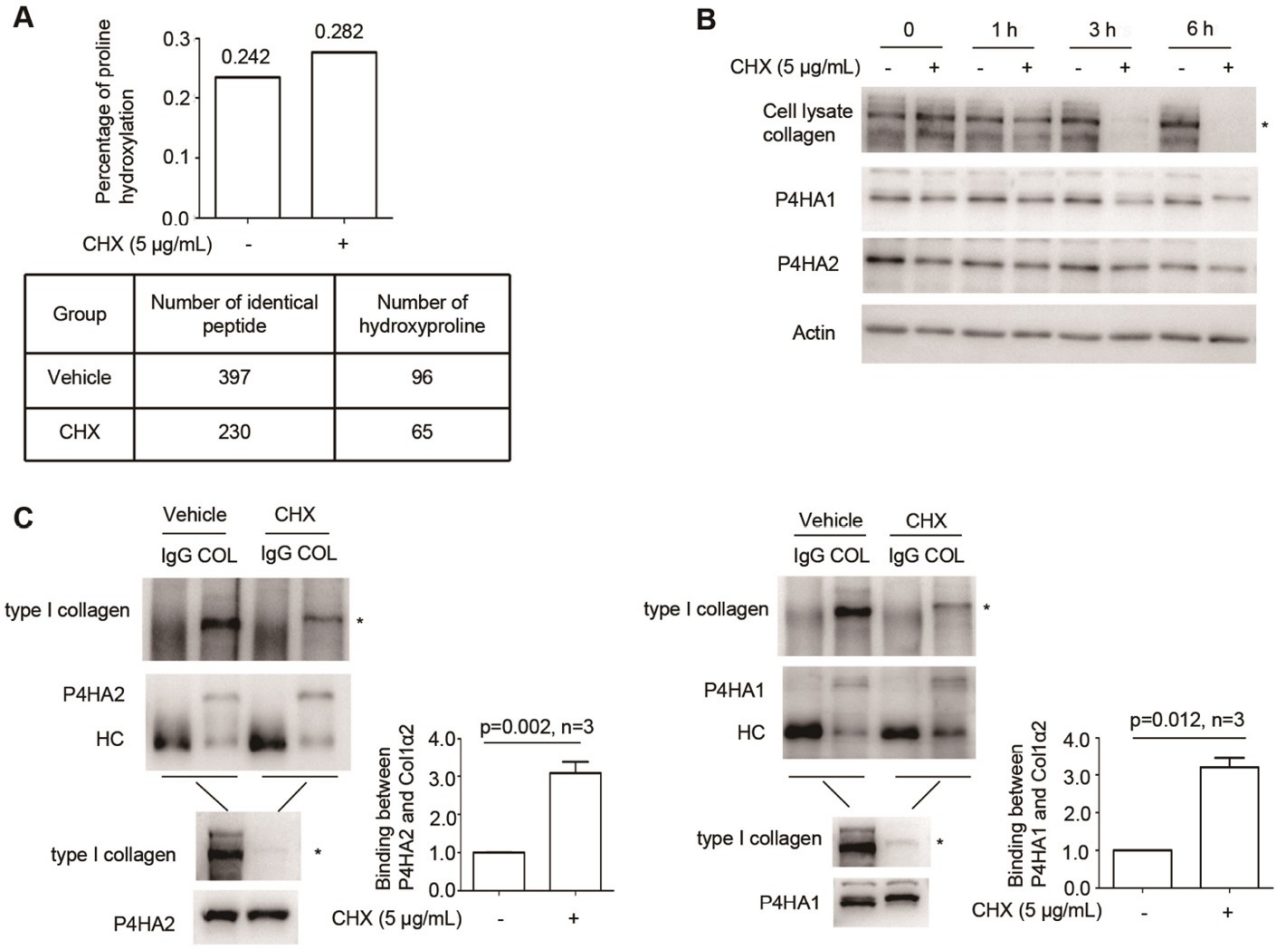
Figure 5 CHX increases type I collagen prolyl hydroxylation by enhancing the binding between P4HA1/2 and type I collagen (A) Percentage of proline hydroxylation on type I collagen with or without CHX treatment. (B) Western blot analysis using type I collagen-specific antibody showed that CHX treatment reduced type I collagen in the ER but did not obviously impact the level of P4HA1/2 protein inside the ER. (C) Co-IP was performed using 2 μg of anti-type I collagen antibody and 1 mL of cell lysate. Copurified col1α2 and P4HA1/2 were detected using the corresponding antibodies. IgG heavy chain (HC) was marked. Statistical analysis showed that the association between col1α2 and P4HA1/2 was enhanced after CHX treatment. The relative ratio between P4HA1/2 and col1α2 in CHX-treated cells was calculated based on the densities of blots assessed by ImageJ software, using the ratio between P4HA1/2 and col1α2 from control cells as a standard, and the ratio between P4HA1/2 and col1α2 in control cells was defined as 1. col1α2: type I collagen α2; COL: anti-type I collagen antibody; * indicates the position of col1α2. Statistical analysis was performed from three independent experiments as indicated. P values are indicated in each panel if available.

We then used mass spectrometry to detect the level of proline hydroxylation in type I collagen purified from culture medium. The results demonstrated that CHX indeed stimulated more hydroxyproline on type I collagen ( Figure 5A and Supplementary Table S1). After treatment with CHX, the level of type I collagen in the cell lysate was decreased ( Figure 5B). In contrast, the level of P4HA1/2 remained relatively stable ( Figure 5B). Since cellular type I collagen and P4HA1/2 are in the ER, this observation suggests that one type I collagen molecule may bind with more P4HA1/2. To assess whether the association between P4HA1/2 and type I collagen is altered when MEFs are treated with CHX, we performed Co-IP using anti-type I collagen-specific antibody. More P4HA1/2 was copurified with type I collagen after MEFs were treated with CHX for 3 h ( Figure 5C). Therefore, a likely mechanism by which CHX activates type I collagen maturation is that each type I collagen can bind with more C-P4Hs, which possess very limited enzymatic activity in the absence of ascorbate [8].

## Discussion

We reported here that CHX treatment altered the ratio of binding between P4HA1/2 and type I collagen by decreasing type I collagen but not P4HA1/2 in the ER. C-P4Hs can catalyze prolyl hydroxylation at a maximal rate for at least a few cycles in the complete absence of ascorbate [8]. Therefore, more P4HA1/2 binding on type I collagen could catalyze more hydroxylation of proline on type I collagen.

As the major isoenzyme in most cells, P4ha1 deficiency leads to embryonic lethality in mice [18]. In contrast, P4HA2 predominates in chondrocytes, and mice deficient in *P4ha2* have no apparent phenotypic abnormalities [ 19– 21] . Both P4HA1 and P4HA2 are expressed in MEFs, but little is known about the substrate preference of these two isoenzymes [22]. The observation that knockout of *P4ha2* reduces much more CHX-stimulated type I collagen maturation than knockout of *P4ha1* suggests that type I collagen hydroxylation in MEFs prefers P4HA2 over P4HA1 under this condition. In contrast, our previous studies have shown that ascorbate stimulates type I collagen secretion mainly via P4HA1, while superoxide-induced type I collagen maturation requires both P4HA1 and P4HA2 [ 11, 17] . Ascorbate is an electron donor that maintains the iron in the Fe ^2+^ state within the C-P4H molecule, thus enabling the C-P4H to repeatedly hydroxylate proline residues [6]. Additionally, ascorbate induces STT3B-dependent N259 glycans on P4HA1 to enhance its binding with type I collagen and facilitate type I collagen maturation [17]. In the absence of ascorbate, superoxide can rescue the enzymatic activity of C-P4Hs by reducing inactive Fe ^3+^ to Fe ^2+^ [23]. Whether superoxide and CHX result in posttranslational modifications on distinct C-P4Hs to affect the substrate preference as ascorbate remains to be investigated.

CHX inhibits protein synthesis by preventing the transfer of activated amino acids from tRNA to growing polypeptides on ribosomal complexes, and as a result, nascent polypeptides remain fixed to ribosomes [24]. However, the P4HA1/2 proteins can be detected in the ER even 6 h post CHX treatment, suggesting that P4HA1/2 are capable of entering the ER several hours after CHX treatment. The mechanism warrants further investigation.

Other than being used as an experimental reagent in molecular biology and cancer biology to inhibit protein synthesis, CHX may cause DNA damage and teratogenesis and have adverse effects on reproduction [ 25– 27] . In addition, CHX can potentiate the apoptotic effects of certain death stimuli [ 28– 36] and block tumor necrosis factor (TNF)-α- or interleukin (IL)-1-induced type I collagen transcription [37]. Since C-P4Hs are the key enzymes in collagen synthesis, they have been regarded as potential therapeutic targets for cancer and fibrosis treatment [ 38, 39] . Clarifying the regulatory mechanism and functions of CHX in type I collagen maturation will facilitate the prevention and treatment of diseases related to aberrant deposition of collagen.

## Supporting information

146Table_S1

146FigS1

## Supplementary data

Supplementary data is available at *Acta Biochimica et Biophysica Sinica* online.
