# Supplementary material for: Cycloheximide promotes type I collagen maturation mainly via collagen prolyl 4-hydroxylase subunit α2: Cycloheximide promotes type I collagen proline hydroxylation
Source: Acta Biochim Biophys Sin (Shanghai). 2022 Dec 20;54(12):1832–40. doi: 10.3724/abbs.2022191 (PMC10157532; doi:10.3724/abbs.2022191)
Supplement: 146Table_S1 [file 146Table_S1.pdf]

Supplementary Table S1. Col 1 α1 and Col 1 α2 containing hydroxyproline and peptide hits by mass spectrometry

Corresponding sites on Col1α1 and Col1 α2 (5'-3')	Sequence of peptide	Group	Number	
			identical peptide	hydroproline
79-104 (Col1α1)	EGECCAFCPEEYVSPNSEDVGVEGPK	Vehicle	1	0
		CHX	1	0
105-113 (Col1α1)	GDPGPQGPR	Vehicle	1	0
		CHX	1	0
161-176 (Col1α1)	SAGVSVPGPMGPSGPR	Vehicle	2	0
		CHX	2	0
210-233 (Col1α1)	GPPGPPGKNGDDGEAGKPGRPGER	Vehicle	2	2
		CHX	2	3
258-266 (Col1α1)	GFSGLDGAK	Vehicle	3	0
		CHX	2	0
360-386 (Col1α1)	GEPGPPGPAGAAGPAGNPGADGQPGAK	Vehicle	3	1
		CHX	1	0
387-404 (Col1α1)	GANGAPGIAGAPGFPGAR	Vehicle	5	4
		CHX	2	2
405-419 (Col1α1)	GPSGPQGPSGPPGPK	Vehicle	0	0
		CHX	1	0
420-431 (Col1α1)	GNSGEPGAPGNK	Vehicle	1	0
		CHX	1	0
438-457 (Col1α1)	GEPGATGVQGPPGPAGEEGK	Vehicle	5	0
		CHX	6	0

462-476 (Col1α1)	GEPGPSGLPGPPGER	Vehicle	3	0
		CHX	2	0
483-494 (Col1α1)	GFPGADGVAGPK.	Vehicle	4	0
		CHX	3	0
510-527 (Col1α1)	GSPGEAGRPGEAGLPGAK	Vehicle	1	0
		CHX	1	0
528-541 (Col1α1)	GLTGSPGSPGPDGK	Vehicle	2	0
		CHX	1	0
542-563 (Col1α1)	TGPPGPAGQDGRPGPAGPPGAR	Vehicle	5	2
		CHX	3	1
564-575 (Col1α1)	GQAGVMGFPGPK	Vehicle	5	1
		CHX	0	0
588-601 (Col1α1)	GLPGPPGAVGPAGK	Vehicle	2	0
		CHX	2	0
588-620 (Col1α1)	GLPGPPGAVGPAGKDGEAGAQGAPGPAGPAGER	Vehicle	5	2
		CHX	1	0
602-620 (Col1α1)	DGEAGAQGAPGPAGPAGER	Vehicle	5	1
		CHX	5	0
621-665 (Col1α1)	GEQGPAGSPGFQGLPGPAGPPGEAGKPGEQGVPGDLGAPGPSGAR	Vehicle	7	5
		CHX	11	18
675-686 (Col1α1)	GVQGPPGPAGPR	Vehicle	3	0
		CHX	2	0
699-722 (Col1α1)	GDTGAPGAPGSQGAPGLQGMPGER	Vehicle	10	7
		CHX	3	1
753-770 (Col1α1)	GLTGPIGPPGPAGAPGDK	Vehicle	3	1

		CHX	1	0
753-785 (Col1α1)	GLTGPIGPPGPAGAPGDKGEAGPSGPPGPTGAR	Vehicle	10	11
		CHX	1	0
771-785 (Col1α1)	GEAGPSGPPGPTGAR	Vehicle	1	0
		CHX	1	0
786-815 (Col1α1)	GAPGDRGEAGPPGPAGFAGPPGADGQPGAK	Vehicle	5	2
		CHX	1	0
792-815 (Col1α1)	GEAGPPGPAGFAGPPGADGQPGAK	Vehicle	2	0
		CHX	4	2
816-824 (Col1α1)	GEPGDTGVK	Vehicle	1	0
		CHX	1	0
816-851 (Col1α1)	GEPGDTGVKGDAGPPGPAGPAGPPGPIGNVGAPGPK	Vehicle	4	3
		CHX	1	1
825-851 (Col1α1)	GDAGPPGPAGPAGPPGPIGNVGAPGPK	Vehicle	5	0
		CHX	2	0
872-892 (Col1α1)	VGPPGPSGNAGPPGPPGPVGK	Vehicle	6	3
		CHX	2	0
900-923 (Col1α1)	GETGPAGRPGEVGPPGPPGPAGEK	Vehicle	4	0
		CHX	2	1
924-947 (Col1α1)	GSPGADGPAGSPGTPGPQGIAGQR	Vehicle	4	0
		CHX	3	3
974-983 (Col1α1)	QGPSGSSGER	Vehicle	1	0
		CHX	1	0
984-1003 (Col1α1)	GPPGPMGPPGLAGPPGESGR	Vehicle	10	6
		CHX	5	3

1004-1015 (Col1α1)	EGSPGAEGSPGR	Vehicle	1	0
		CHX	1	0
1004-1022 (Col1α1)	EGSPGAEGSPGRDGAPGAK	Vehicle	2	0
		CHX	2	0
1023-1051 (Col1α1)	GDRGETGPAGPPGAPGAPGAPGPVGPAGK	Vehicle	5	5
		CHX	2	0
1026-1051 (Col1α1)	GETGPAGPPGAPGAPGAPGPVGPAGK	Vehicle	5	4
		CHX	3	2
1052-1073 (Col1α1)	NGDRGETGPAGPAGPIGPAGAR	Vehicle	3	0
		CHX	1	0
1056-1073 (Col1α1)	GETGPAGPAGPIGPAGAR	Vehicle	3	0
		CHX	3	0
1101-1130 (Col1α1)	GFSGLQGPPGSPGSPGEQGPSGASGPAGPR	Vehicle	4	5
		CHX	2	3
1131-1141 (Col1α1)	GPPGSAGSPGK	Vehicle	2	0
		CHX	1	0
1131-1157 (Col1α1)	GPPGSAGSPGKDGLNGLPGPIGPPGPR	Vehicle	2	0
		CHX	3	5
1142-1157 (Col1α1)	DGLNGLPGPIGPPGPR	Vehicle	4	2
		CHX	3	1
1207-1214 (Col1α1)	ADDANVVR	Vehicle	2	0
		CHX	5	0
1215-1225 (Col1α1)	DRDLEVDTTLK	Vehicle	6	0
		CHX	5	0
1226-1235 (Col1α1)	SLSQQIENIR	Vehicle	4	0

		CHX	2	0
1260-1277 (Col1α1)	SGEYWIDPNQGCNLDAIK	Vehicle	3	0
		CHX	1	0
1278-1299 (Col1α1)	VYCNMETGQTCVFPTQPSVPQK	Vehicle	6	0
		CHX	4	0
1300-1308 (Col1α1)	NWYISPNPK	Vehicle	3	0
		CHX	2	0
1312-1345 (Col1α1)	HVWFGESMTDGFPFEYGSEGSDPADVAIQLTFLR	Vehicle	1	1
		CHX	1	2
1361-1374 (Col1α1)	NSVAYMDQQTGNLK	Vehicle	2	0
		CHX	2	0
1361-1375 (Col1α1)	NSVAYMDQQTGNLKK	Vehicle	5	0
		CHX	3	0
1376-1388 (Col1α1)	ALLLQGSNEIELR	Vehicle	48	0
		CHX	30	0
1395-1413 (Col1α1)	FTYSTLVDGCTSHTGTWGK	Vehicle	13	0
		CHX	4	0
1426-1453 (Col1α1)	LPIIDVAPLDIGAPDQEFGLDIGPACFV	Vehicle	3	0
		CHX	4	1
231-240 (Col1α2)	VGAPGPAGAR	Vehicle	1	0
		CHX	1	0
241-270 (Col1α2)	GSDGSVGPVGPAGPIGSAGPPGFPGAPGPK	Vehicle	3	1
		CHX	2	1
271-288 (Col1α2)	GELGPVGNPGPAGPAGPR	Vehicle	2	1
		CHX	1	0

334-348 (Col1α2)	GIPGPAGAAGATGAR	Vehicle	1	0
		CHX	2	1
349-360 (Col1α2)	GLVGEPGPAGSK	Vehicle	1	0
		CHX	1	0
388-405 (Col1α2)	GSPGEAGSAGPAGPPGLR	Vehicle	2	0
		CHX	2	2
457-470 (Col1α2)	GLPGSPGNVGPSGK	Vehicle	2	0
		CHX	1	0
471-492 (Col1α2)	EGPVGLPGIDGRPGPIGPAGPR	Vehicle	3	1
		CHX	1	0
493-504 (Col1α2)	GEAGNIGFPGPK	Vehicle	3	1
		CHX	1	0
505-516 (Col1α2)	GPSGDPGKPGER	Vehicle	1	0
		CHX	1	0
526-549 (Col1α2)	GAPGPDGNNGAQGPPGPQGVQGGK	Vehicle	3	2
		CHX	1	1
580-594 (Col1α2)	GLPGEFGLPGPAGPR	Vehicle	3	0
		CHX	1	0
598-615 (Col1α2)	GTPGESGAAGPSGPIGSR	Vehicle	2	0
		CHX	1	0
616-651 (Col1α2)	GPSGAPGPDGNKGEAGAVGAPGSAGASGPGGLPGER	Vehicle	4	3
		CHX	3	3
628-651 (Col1α2)	GEAGAVGAPGSAGASGPGGLPGER	Vehicle	2	0
		CHX	2	1
652-660 (Col1α2)	GAAGIPGGK	Vehicle	2	0

		CHX	1	0
682-699 (Col1α2)	GIPGAVGAPGPAGASGDR	Vehicle	4	0
		CHX	2	1
682-714 (Col1α2)	GIPGAVGAPGPAGASGDRGEAGAAGPSGPAGPR	Vehicle	3	1
		CHX	1	0
700-714 (Col1α2)	GEAGAAGPSGPAGPR	Vehicle	1	0
		CHX	1	0
801-821 (Col1α2)	TGPPGPSGIAGPPGPPGAAGK	Vehicle	4	3
		CHX	5	4
837-852 (Col1α2)	TGETGASGPPGFVGEK	Vehicle	2	0
		CHX	1	0
837-885 (Col1α2)	TGETGASGPPGFVGEKGPSGEPGTAGAPGTAGPQGLLGAPGILGLPGSR	Vehicle	3	6
		CHX	1	2
853-885 (Col1α2)	GPSGEPGTAGAPGTAGPQGLLGAPGILGLPGSR	Vehicle	2	1
		CHX	1	0
889-912 (Col1α2)	GLPGIAGALGEPGPLGISGPPGAR	Vehicle	6	3
		CHX	1	0
913-932 (Col1α2)	GPPGAVGSPGVNGAPGEAGR	Vehicle	5	4
		CHX	1	0
955-980 (Col1α2)	GYPGSIGPTGAAGAPGPHGSVGPAGK	Vehicle	3	0
		CHX	2	0
985-1002 (Col1α2)	GEPGPAGSVGPVGAVGPR	Vehicle	3	0
		CHX	1	0
1003-1011 (Col1α2)	GPSGPQGIR	Vehicle	2	0
		CHX	1	0

1030-1059 (Col1α2)	GYSGLQGLPGLAGLHGDQGAPGPVGPAGPR	Vehicle	5	0
		CHX	1	0
1060-1070 (Col1α2)	GPAGPSGPVGK	Vehicle	1	0
		CHX	1	0
1074-1086 (Col1α2)	SGQPGPVGPAGVR	Vehicle	1	0
		CHX	1	0
1138-1146 (Col1α2)	DYEVDATLK	Vehicle	2	0
		CHX	1	0
1147-1162 (Col1α2)	SLNNQIETLLTPEGSR	Vehicle	3	0
		CHX	1	0
1174-1198 (Col1α2)	LSHPEWNSDYYWIDPNQGCTMDAIK	Vehicle	4	1
		CHX	2	0
1199-1220 (Col1α2)	VYCDFSTGETCIQAQPVNTPAK	Vehicle	2	0
		CHX	2	0
1280-1294 (Col1α2)	NSIAYLDEETGSLNK	Vehicle	6	0
		CHX	3	0
1295-1313 (Col1α2)	AVLLQGSNDVELVAEGNSR	Vehicle	35	0
		CHX	12	0
1314-1325 (Col1α2)	FTYSVLVDGCSK	Vehicle	5	0
		CHX	3	0
1364-1372 (Col1α2)	VEVGPVCFK	Vehicle	2	0
		CHX	1	0
