# Supplementary material for: Cycloheximide promotes type I collagen maturation mainly via collagen prolyl 4-hydroxylase subunit α2: Cycloheximide promotes type I collagen proline hydroxylation
Source: Acta Biochim Biophys Sin (Shanghai). 2022 Dec 20;54(12):1832–40. doi: 10.3724/abbs.2022191 (PMC10157532; doi:10.3724/abbs.2022191)
Supplement: 146FigS1 [file 146FigS1.pdf]

Supplemental Figure 1

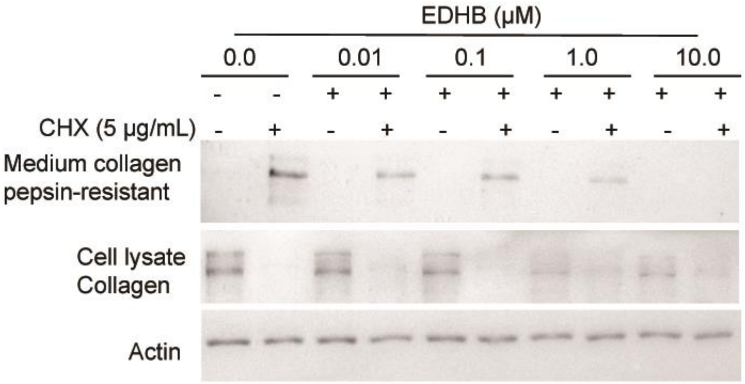

Supplementary Figure S1. CHX-induced type I collagen maturation depends upon the activity of C-P4Hs MEFs were treated with different concentrations of EDHB in the presence or absence of 5 µg/mL CHX. Western blot analysis using type I collagen specific antibody showed that EDHB used at the concentration of 10 µM completely abolished pepsin-resistant type I collagen in the culture medium.
